# Depression symptoms among adolescents in Morocco: a school-based cross-sectional study

**DOI:** 10.11604/pamj.2023.44.147.36713

**Published:** 2023-03-27

**Authors:** Marouane Moustakbal, Souad Belabbes Maataoui

**Affiliations:** 1 Biochemistry, Neurosciences, Natural Resources and Environment Laboratory, Sciences and Technologies Faculty, Hassan 1^st^ University, Settat, Morocco

**Keywords:** Depressive symptoms, mental health, daytime sleepiness, academic achievement, adolescents, Morocco

## Abstract

**Introduction:**

depression in adolescents is a major health condition that can interfere with daily life, lead to suicidal thoughts and behavior, and affect a person throughout life. However, studies about adolescents´ depression in Morocco are scarce. The aim of this study was to determine the prevalence of depression symptoms among in-school adolescents from the region of Settat-Morocco, as well as assessing its relation to daytime sleepiness and poor academic achievement.

**Methods:**

the researchers conducted a cross-sectional school-based study. The sample included participants aged 12-20 years, in either urban or rural areas. We selected 722 students through a proportionate stratified sampling procedure. The participants responded to multiple questionnaires that included the Patient Health Questionnaire-9, the Epworth Sleepiness Scale, a questionnaire assessing several sociodemographic and socioeconomic variables, and finally an academic achievement questionnaire. We analyzed the collected data using descriptive statistical methods, χ2 test, and odds ratios.

**Results:**

forty-four point seven percent (44.7%) of the respondents had “moderately severe” to “severe depression symptoms” and 32.5% of the sample suffered from excessive daytime sleepiness. Nineteen point nine percent (19.9%) of the total sample reported having a poor academic achievement. Significant predictors for depression symptoms included “female gender” (OR = 2.06; p-value < 0.001), “having divorced parents” (OR = 6.00; p-value < 0.001), “poor academic achievement” (OR = 5.03; p-value < 0.001), and “excessive daytime sleepiness” (OR = 2.30; p-value = 0.002).

**Conclusion:**

this study provides important information about Morocco adolescents´ depression symptoms. These findings can help in implementing school-based mental and sleep health programs that target the promotion of mental wellness, prevention of mental health problems, and reduction of adolescent suicide risk.

## Introduction

Adolescence is a critical period marked by hormonal, physical, psychological, and social changes [1]. At present, adolescents aged between 10 and 20 years represent about one-fifth of the Morocco population and are an important and dynamic part of the fabric of society [2]. Addressing the health and well-being needs of adolescents is a fundamental goal to ensure that they thrive and can achieve their full potential [3]. Promoting psychological well-being and protecting adolescents from adverse experiences and risk factors that could affect their development is critical for achieving this goal [4]. Mental health conditions are the leading impairment affecting adolescents´ development, including educative and social achievements [5]. Reviews of literature on the prevalence of depression among adolescents report high percentages of depressive symptoms [6]. Adolescent depression is often unrecognized and untreated, despite evidence that the duration of untreated depressive illness is a major factor predicting recurrence in adult life [7]. Signs and symptoms of depression include changes in attitude, behavior, and sleep that can cause severe distress and problems at school, at home, at social activities, or in other areas of life [8]. An early report of Moroccan adolescents estimated the prevalence of major depressive disorder at 26.5% [9]. Research on adolescents´ self-injury has found that some youth who develop depression often engaged in significant self-harm with signs of suicidal ideation [10]. In addition, excessive daytime sleepiness (EDS) is a complaint frequently encountered in individuals with major depression [11,12]. Daytime sleepiness is an important public health issue that affects the performance, cognitive functions, and mental condition of school children and causes them to have difficulties in concentrating; to suffer from fatigue; and to experience irritability, anxiety, and depression [13]. A one-year prospective study among adolescents showed evidence of a bidirectional association between excessive daytime sleepiness and depressive symptoms [14]. Furthermore, poor academic achievement and inconsistent school attendance are predictors of emerging or existing mental health problems during childhood and adolescence [15,16]. Academic achievement, however, is notoriously difficult to quantify, and the measures commonly used in school settings, grades and test scores suffer from inherent limitations regarding objectivity (e.g., teacher bias), reliability and validity [17]. Grades and test scores are designed to capture accumulated knowledge and thus may be less sensitive to contemporaneous differences in middle and high school settings [18]. Numerous studies suggest that non-test score outcomes may yield important insights into students´ experiences at school [19,20]. Skills such as executive function, grit, and perseverance may not be well-reflected in test scores but may ultimately affect students´ long-term success [21].

Measures of attendance and other non-cognitive outcomes can capture both student engagement and non-cognitive skills (such as persistence and executive function) [21,22]. Absenteeism might also reflect differences in health that may be due to changes in sleeping patterns [21,23]. Lateness, which measures a more nuanced level of punctuality compared with absenteeism and represents a non-cognitive skill that may be particularly relevant in the labor market (e.g., the ability to show up to work on time). While student scores might be an important outcome in itself, it may not be a good indicator of engagement, given that individuals who have high scores may not necessarily be more engaged. Many studies of student emotional engagement treat it as a predictor of academic achievement, inferring that being disengaged or disaffected from school causes poor academic achievement. However, the theoretical literature argues that it is a low achievement that causes students to withdraw from school, or that engagement and academic achievement go hand-in-hand [24,25]. Taken together, the existing evidence across a range of studies suggests that both cognitive and noncognitive skills have equal importance in students´ academic achievement as a multidimensional concept; and therefore, should be both incorporated as measures of success in education.

Whereas most studies of depression symptoms and associated demographic and clinical factors have been conducted on Western children, little is known about the prevalence of depression symptoms in a North African context, where school children may experience more social and academic pressure. Additionally, children´s sleeping behavior is rarely specifically tested as an associated factor in most modern studies focused on adolescents in Morocco. Consequently, sleep problems, such as excessive daytime sleepiness and its relation to depression symptomology in adolescent populations, remain largely unexplored. Considering the above, the first aim of this article was to investigate the prevalence of depressive symptoms and excessive daytime sleepiness in a sample of school-aged adolescents from the central region of Morocco. The second aim was to describe how some demographic and clinical factors are associated with depressive symptomatology. We hypothesized that adolescents with depressive symptoms would exhibit greater daytime sleepiness and poorer academic performance.

## Methods

**Study design:** the researchers carried out a school-based, observational cross-sectional study. Accessing young people in schools for research studies is one of the most resource-efficient methods. This approach permits the collection of data all at once and, thus, the study of multiple outcomes, which lower the time and cost of the study. Cross-sectional design is the most relevant design when assessing the prevalence of disease, attitudes, and knowledge among patients. This design provides a valuable tool for identifying associations that can then be more rigorously studied, using a cohort study or randomized controlled study.

**Setting:** we carried out the study between December 2019 and February 2020 in the Settat province, north center of Morocco. Settat is one of the 7 provinces of the Casablanca-Settat administrative region. Settat is an urban center located at latitude 33° 00' 00" N and longitude 7° 37' 00" W. The province has a total area of approximately 7125 km^2^ and a population of 634,184 based on the 2014 census [26]. Settat is subdivided into 44 municipalities with >90% composed of agrarian communities. According to the regional directorate of education, there was a total of 23755 in-school adolescents, studying in 79 registered secondary schools, of which 28 are urban and 51 are rural.

**Study population:** participants selected in the study were all consenting students from both middle and high schools, aged between 12 and 20 years. The regional directorate of education in Settat provided the researchers with a list of all secondary schools and the student population in the province, which at the moment of the study was (N) = 23755 students. The population database regrouped all students in the region into two subgroups: students in urban schools and students in rural schools.

### Sampling

Sample size calculation was done using a free online tool, G-Power software (version 3.1). G-Power software application is used to perform a priori power analysis for studies. It is easy to use for calculating sample size and power for various statistical methods (F, t, χ2, Z, and exact tests) [27]. A confidence level of 0.95 and a power of 0.80 was assumed. The expected proportion of depression symptoms among the participants was fixed at 0.4, based on previous studies [28,29]. The power analysis indicated that the total sample size needed for the study is around n = 750. To ensure a balanced sample of both rural and urban adolescents, we designed a proportionate stratified sampling procedure to include participants based on their proportionate allocation in the actual population. This type of sampling technique has a high statistical precision compared to simple random sampling and guarantees better coverage of the population and less selection bias. The cumulative population per stratum was divided by the total population and multiplied by the estimated sample size to determine the number of students that would be required to take part per stratum. Based on this calculation, seven urban and two rural schools were selected by probability proportional to size. In strata 1, we included 7 randomly selected urban schools. In strata 2, we included 2 randomly selected rural schools. Then, we randomly drew 800 adolescents from student´s lists of the 9 selected schools to take part in the study.

**Measures:** the researchers developed the tool in Arabic, the students´ native language, which included four questionnaires that were pretested prior to data collection. The questionnaires were the following: patient health questionnaire 9 (PHQ): a 9-item self-report questionnaire which aims to assess the severity of depression [17]. The respondent expresses their agreement or disagreement with each statement on a 4-point scale (0 = not at all; 1 = several days; 2 = more than half the days; and 3 = nearly every day). The total score can range from 0 to 27, with higher scores indicating greater severity of depression symptoms. A global score over 10 indicates moderate-to-severe depression symptoms. In the current study, we used the Arabic version of the questionnaire. The questionnaire´s internal consistency was a = 0.85.

Epworth Sleepiness Scale (ESS): a self-administered, eight-item, well-validated, and widely used subjective sleepiness scale [30]. The questionnaire asks the respondent to score the likelihood of falling asleep in eight different situations during waking hours. Scores on the Epworth Sleepiness Scale range from 0 to 24, with higher scores indicating a greater likelihood of sleepiness: Scores <10 indicate less severe sleepiness, and scores > 10 indicate clinically significant sleepiness [31]. Demographic questionnaire: Participants responded to a questionnaire about demographic variables such as gender, age, parents´ status and number of siblings. In order to assess participant´s hobbies, the questionnaire included three items, which asked the participants whether they read books, used social media or participated in sports activities. Academic achievement questionnaire: The researchers measured school achievement during the last three months preceding the research. We used a questionnaire comprising four items measuring both cognitive and noncognitive skills: The grade point average as a measure of student performance, school importance as a measure of emotional engagement, lateness as a measure for commitment, and absenteeism as a measure for non-disruptive behavior.

The grade point average (GPA) in the Moroccan educational system uses a 20-point grading scale. On this basis, the questionnaire comprised a 4-point Likert scale devising the 20-point grading scale to 4 levels: 0 and less than 5 (low), 5 and less than 10, 10 and less than 15, between 15 and 20 (excellent). The second item measured school importance on a 4-point Likert scale from “Extremely unimportant” to “Extremely important”. Lateness and absenteeism were each measured on a 4-point Likert scale from always to never. The researchers calculated the global score by adding the scores of all four items. The total score ranged from 0 to 16: Scores equal to 16 indicated excellent academic achievements, scores between 8 and 15 indicated average academic achievement, and scores less than 8 indicated poor academic achievements.

**Data collection:** the researchers conducted the study at the convenience of the schools. We reminded the participants that their participation in this study was voluntary. After giving instructions, 30 minutes were allotted for the students to complete the questionnaires (most participants completed the questionnaire in 20~30 min).

**Outcome variable:** the outcome variable of interest was depressive symptomology. We calculated a summed score from the PHQ-9 questionnaire, with higher scores indicating more depressive symptomology (range = 0-27). PHQ-9 scores were categorized into a dichotomous variable using the previously established cut-off value: 0-10 = no depressive symptoms, 11-27 = depressive symptoms).

**Co-variables:** individual characteristics were measured by gender (male/female), age groups (12-14/15-17/18-20), region (rural adolescent/urban adolescent), parental status (married/divorced/one or both parents deceased), number of siblings (no siblings/one or two/over two). Measured personal habits included three dichotomous variables with yes or no responses (reading books/use of social apps/playing sports). We recoded academic achievement using the previously established cut-off values into three categories (low/average/excellent). We categorized the Epworth Sleepiness Scale scores into a dichotomous variable using the previously established cut-off value: a score of < 11 is a low risk for sleepiness and =11 is a high risk.

**Statistical analysis:** the researchers carried out the statistical analyses with the IBM SPSS (IBM Corp. 2012. IBM SPSS Statistics for Windows, version 21.0. NY, EUA). The study used frequencies and percentages to get the prevalence and general characteristics of the population sampled. We described age as a numerical variable using means and standard deviations. Pairwise deletion was used to address missing data. Cross-tabulations (contingency tables) and the two-tailed Chi-square test of independence were used to examine the relationship between independent categorical variables (demographic, academic, and clinical characteristics) and the outcome (depressive symptomology). The study included variables significant at the 5% level, in a logistic multivariable analysis model which quantified the relationship between the various covariates and the outcome variable as odd ratios.

**Ethical considerations:** this is a study of public behavior that was purely observational and was only involved in the collection and analysis of data, as no intervention on the human participants was needed. Nevertheless, in respect to we sought ethical approval and authorization from the educational directorate in the study's region. After internal deliberation, we were cleared to perform the study but in complete oversight of the data collection process by the parental association in every school visited. For any questions regarding this process, please contact the Settat regional directorate of education on (+212) 5234-02704 or at dp.settat@gmail.com. Informed consent was obtained from the students verbally and written in every administered questionnaire. The researchers included no personal identifiers in the questionnaire. We maintained anonymity and confidentiality throughout data collection, storage, and analysis.

**Funding statement:** this research did not receive any specific grant from funding agencies in the public, commercial, or not-for-profit sectors. The authors received no financial support for the research, authorship, and publication of this article.

**Data availability:** the data that supports the findings of this study are available on request from Marouane Moustakbal, the corresponding author.

## Results

The researchers invited 800 middle and high school students to participate in the study. Six hundred and seventy-six (676) participants fulfilled the study inclusion criteria and completed both the PHQ-9 and the ESS, corresponding to a response rate of 84.5%. Gender identification was female (56.1%) and male (43.9%). The mean age (±SD) was 15.64 ± 1.68 years, with 206 (30.5%) in the early adolescent age group (10-14 years), 368 (54.4%) in the middle adolescent age group (15-17 years), and 102 (15.1%) in the late adolescent age group (18-20 years). The sample included students from different regions, 349 (51.6%) were from rural regions, while 327 (48.4%) of the respondents were from urban regions. Clinically, 32.5% of the sample (N = 220) suffered from excessive daytime sleepiness. Forty-four point seven percent (44.7%) of the sample experienced moderate-severe symptoms of depression. In terms of academic achievement, 19.0% of the total sample reported poor academic achievement during the last three months preceding the research. We reported all the socio-demographic, academic achievement and clinical characteristics of the participants in Table 1.

**Table 1:** socio-demographic characteristics, academic achievement and clinical characteristics

Age (M; SD)	15.64	1.68	
	**Count**	**(%)**	**Missing Data**
**Gender (Female)**			
Male	297	43.9	0
Female	379	56.1	
**Region**			
Rural	349	51.6	0
Urban	327	48.4	
**Age in groups**			
12 - 14	206	30.5	0
15 - 17	368	54.4	
18 - 20	102	15.1	
**Parents status**			
Married	606	90.7	8
Divorced	23	3.4	
One parent Deceased	39	5.8	
**Number of siblings**			
0	23	3.4	0
1 - 2	250	37.0	
≥3	403	59.6	
**Reading books**			
Yes	201	29.8	1
No	474	70.2	
**Use of social apps**			
Yes	428	63.4	1
No	247	36.6	
**Playing sports**			
Yes	65	9.6	1
No	610	90.4	
**Academic achievement**			
Excellent	52	8.4	59
Average	448	72.6	
Poor	117	19.0	
**Clinical characteristics**			
Excessive Daytime sleepiness	220	32.5	0
Moderate to severe symptoms of depression	302	44.7	0
Means (M); Standard Deviations (SD)			

Compared to those without depression symptoms, most adolescents with depression symptoms were from female gender [49.9%; vs. 38.0%; χ2 (1) = 9.41, p = 0.002] and were from rural regions [49.6%; vs. 39.4%; χ2 (1) = 6.99, p = 0.008]. The prevalence of symptoms consistent with depressive disorder was higher among older respondents (15-17 and 18-20) compared to those from younger age (12-14) [50.8%; vs. 49.0%; vs. 31.6%; χ2 (2) = 20.74, p < 0.001]. Also, the researchers observed a relationship between the occurrence of depressive symptoms and the parental status of the respondent: The highest proportion of depressive symptoms is among respondents with divorced parents and the lowest proportion is among those with married parents [73.9%; vs. 42.7%; χ2 (2) = 11.05, p = 0.004]. Respondents who did not take part in any sporting activities had a higher proportion of depressive symptoms when compared with those who took part in sporting activities [45.9%; vs. 32.3%; χ2 (1) = 4.39, p = 0.036].

In terms of academic characteristics, depressive symptoms were also statistically correlated with academic achievements. The highest proportion of depressive symptoms was among students with poor academic achievement and the lowest proportion was among those with excellent academic achievement [62.4%; vs. 26.9%; χ2 (1) = 24.04, p < 0.001]. In terms of clinical characteristics, adolescents with depressive symptoms reported high risk for sleepiness compared to those without depressive symptoms [55.9%; vs. 39.3%; χ2(1) = 16.65, p < 0.001]. The results in Table 2 show the association between depression symptoms status and each of socio-demographic characteristics, academic achievement and daytime sleepiness. The researchers performed a binary logistic regression analysis to investigate the effects of factors associated with depression symptoms. The logistic regression model was statistically significant, χ2(12) = 89.701, p < 0.001. In this study, the model explained 18.0% (Nagelkerke R2) of the variance in depression symptomology and correctly classified 66.4% of cases. Table 3 summarises the analysis results.

**Table 2:** Chi-square test for association between depression symptoms status and each of socio-demographic characteristics, academic achievement and daytime sleepiness

Variable	Depressed (%)	Not depressed (%)	Total (%)	X^2^ value	Dfg^2^	P value (a = 0.05)
**Gender**						
Male	113 (38.0)	184 (62.0)	297 (100)	9.41	1	0.002*
Female	189 (49.9)	190 (50.1)	379 (100)			
**Region**						
Rural	173 (49.6)	176 (50.4)	349 (100)	6.99	1	0.008*
Urban	129 (39.4)	198 (60.6)	327 (100)			
**Age in groups**						
12 - 14	65 (31.6)	141 (68.4)	206 (100)	20.74	2	0.000*
15 - 17	187 (50.8)	181 (49.2)	368 (100)			
18 - 20	50 (49.0)	52 (51.0)	102 (100)			
**Parents status**						
Married	259 (42.7)	347 (57.3)	606 (100)	11.05	2	0.004*
Divorced	17 (73.9)	6 (26.1)	23 (100)			
One parent Deceased	22 (56.4)	17 (43.6)	39 (100)			
**Number of siblings**						
0	12 (52.2)	11 (47.8)	23 (100)	0.64	2	0.725
1 - 2	113 (45.2)	137 (54.8)	250 (100)			
≥3	177 (43.9)	226 (56.1)	403 (100)			
**Reading books**						
Yes	74 (36.8)	127 (63.2)	201 (100)	7.01	1	0.008*
No	227 (47.9)	247 (52.1)	474 (100)			
**Use of social apps**						
Yes	205 (47.9)	223 (52.1)	428 (100)	5.17	1	0.023*
No	96 (38.9)	151 (61.1)	247 (100)			
**Playing sports**						
Yes	21 (32.3)	44 (67.7)	65 (100)	4.39	1	0.036*
No	280 (45.9)	330 (54.1)	610 (100)			
**Academic achievement**						
Poor	73 (62.4)	44 (37.6)	117 (100)	24.04	2	0.000*
Average	183 (40.8)	265 (59.2)	448 (100)			
Excellent	14 (26.9)	38 (73.1)	52 (100)			
**Daytime sleepiness**						
Low risk for sleepiness	179 (39.3)	277 (60.7)	456 (100)	16.65	1	0.000*
High risk for sleepiness	123 (55.9)	97(44.1)	220 (100)			

**Table 3:** regression of depression symptoms status on significant correlates

Variable	Odds ratio	95% CI OR	P value
**Gender**			
Female	2.06	1,41 - 3,01	0.000*
Male	1.00	-	
**Region**			
Rural	1.34	0.92 - 1.95	0.128
Urban	1.00	-	
**Age in groups**			
15 - 17	1.71	1.10 - 2.67	0.017*
18 - 20	1.44	0.80 - 2.60	0.229
12 - 14	1.00	-	
**Parents status**			
Divorced	6.00	1.76 - 20.49	0.004*
One parent Deceased	1.99	0.92 - 4.30	0.079
Married	1.00	-	
**Reading books**			
No	1.19	0.81 - 1.76	0.373
Yes	1.00	-	
**Use of social apps**			
Yes	1.32	0.91 - 1.93	0.140
No	1.00	-	
**Playing sports**			
No	1.25	0.67 - 2.33	0.477
Yes	1.00	-	
**Academic achievement**			
Poor	5.03	2.22 - 11.38	0.000*
Average	2.05	1.03 - 4.10	0.041*
Excellent	1.00	-	
**Daytime sleepiness**			
High risk for sleepiness	2.30	1.58 - 3.34	0.000*
Low risk for sleepiness	1.00	-	

* Significant at 5% level of significance/95% Confidence interval

Female respondents were 2.06 times more likely to exhibit depression symptoms than male respondents (95% CI, 1,41 - 3,01; p < 0.001). Adolescents aged 15-17 were 1.71 times more likely to exhibit depression symptoms than their counterparts from other age groups (95% CI, 1.10 - 2.67; p = 0.017). Regarding parents´ status, having divorced parents was an important risk factor for depression symptoms (OR = 6.00; [95% CI, 1.76 - 20.49; p < 0.001). Respondents with low academic achievement were 5.03 times more likely to exhibit depression symptoms than respondents with excellent academic achievement (95% CI, 2.22 - 11.38; p < 0.001). Daytime sleepiness had a highly significant association with the existence of depression, as those who suffered from excessive daytime sleepiness were more likely to develop depression symptoms than those with normal sleep (OR = 2.30; 95% CI, .58 - 3.34; p < 0.001).

## Discussion

The current study aimed to estimate the frequencies of depressive symptoms and sleep deprivation in a Moroccan sample of adolescents. The prevalence of depressive symptoms among in-school adolescents was 44.7%. This is consistent with a report by the Moroccan Ministry of Health stating that 48.9% of Moroccan adolescents had a problem with insomnia, anxiety, and depression [28]. This result is slightly higher than the findings of Eloirdi *et al*. (2014), who reported a prevalence of 40% for moderate-to-severe symptoms of depression among Moroccan adolescents using the Mini International Neuropsychiatric Interview (MINI) [29]. In comparison, 13.3% of the US population aged 12 to 17 had at least one major depressive episode during a year, according to the 2017 National Survey on Drug Use and Health [32]. The high proportion of depression among Moroccan adolescents compared to adolescents from a developed country like the US could be explained by several factors linked to social and cultural differences, as adolescents in low- and middle-income countries suffer the greatest exposure to environmental and social risk factors such as poverty, violence, natural disasters, and unavailability of psychological treatments [33]. A study among Moroccan adolescents found that 90% experienced violent discipline and 88% of those interviewed reported experiencing psychological aggression [34]. In addition, the current study showed a higher prevalence of depressive symptoms among females compared to males. This is in line with different studies that showed a preponderance of depression among females [35,36]. The results within this study context may be exacerbated by the social pressure, cultural constraints, and religious forces faced more by young Moroccan girls compared to their male counterparts [37].

The study also showed that students from rural regions are more commonly associated with depressive symptoms compared to students from urban areas. These findings are consistent with previous reports of a strong association between having a rural vs. urban background and depression among adolescents [38,39]. We may ascribe this in Morocco to a higher rate of poverty in rural areas and precarious conditions of living, with most rural families, constituted of large numbers of people living in typically small dwellings [40]. Different studies show that adolescents growing up in impoverished conditions reported higher levels of depression than their non-impoverished counterparts [41,42]. The effect of the age difference between rural and urban areas may have also confounded the results in this sample, as the proportion of students from older age was higher in rural areas compared to urban areas. As observed by Supa and Karlin in a nationally representative study on school-going adolescents in Morocco, older adolescents were almost twice more likely to experience depression symptoms as younger ones [43]. Studies in the literature established that the prevalence of depression symptoms dramatically increases with age among adolescents [44,45]. The association between parental status and depression symptoms among adolescents found in the present study is consistent with the results of a study by Bastaits *et al*. which evaluated the effect of parental divorce on adolescents´ subjective well-being [46]. Bastaits *et al*. concluded that adolescents having divorced parents have a higher likelihood of depressive feelings than adolescents living with still-married parents [46]. Likewise, many studies have shown the poorer quality of life and particularly higher prevalence of depression among bereaved children and adolescents, even years after losing a parent [47-49].

The current study showed a significant negative association between reading books and depressive symptomology. Previous researches have established that books can have a small to moderate positive effect on child mental health, especially self-help books [50]. This result may also be explained by the fact that most of the adolescents who read books are from higher social class and fewer people among the underprivileged poor population read, with only 2 minutes of reading time in a day among most Moroccan adolescents, as established in a study by the Moroccan High Commission for Planning [51]. In this study, adolescents´ use of social media was associated with higher levels of depressive symptoms. Various studies showed that adolescents with high use of social media had higher rates of depression and anxiety than did light users, but the evidence is not conclusive [52,53]. As in previous research, this study showed that there was a negative association between physical activity and the severity of depression symptoms [54,55]. These results may also explain the gender difference in the proportion of depression symptoms, as Moroccan male adolescents are more likely to be active compared to female adolescents who spent more time in moderate-intensity physical activity at home [56].

When examining the impact of academic achievement in the school context, the study associated low academic achievement with increased depressive symptoms. Similarly, previous studies provide empirical support for this direction of the relation, showing that children and adolescents who reported lower levels of academic achievement reported greater depressive symptoms over time [57]. Also, the relation between the two variables may be bidirectional, especially for girls, as shown in a longitudinal study of Dutch preadolescents [58]. Concerning excessive daytime sleepiness, our results are in line with several studies using the Epworth Sleepiness Scale, which concluded that EDS significantly predicted depression symptomology [11,12,59]. Insufficient sleep and daytime sleepiness seem to have a robust relationship with mood dysregulation and depressive symptoms [60].

Our findings should be interpreted in light of certain limitations. One potential limitation of the study is that all participants were recruited from one geographical area. Although the findings may not generalise across the country, the sample demographics and the prevalence of depression symptoms are consistent with national statistics. Additionally, the study is cross-sectional, and the data collected is self-reported. Collected data may be more susceptible to recall bias, also results of the current study cannot speak to causality, and full temporal order cannot be demonstrated. Although these findings provide data that confirm a considerably high level of depression symptoms among Moroccan adolescents, such studies cannot replace full clinical assessment but should serve as a warning to the high proportion of teens at risk of depression and even suicidal ideation, especially among girls, as Moroccan women have the highest percentage of total suicide in the Middle East and North Africa [61].

## Conclusion

Our study offers several avenues and implications. At the time of this study, there are no facilities in Moroccan schools for proper screening and evaluation of adolescents for major depressive disorders. Adequate systems should be implemented in place to ensure accurate screening of depression at the Moroccan schools. Routine screening for depression can help in the identification of adolescents at risk and should be followed by confirmation of the diagnosis by a psychiatrist. Practically, the recruitment of school social workers should be an important goal for Moroccan educational authorities with an emphasis on school-based mental health programs that can focus on promoting mental wellness, preventing mental health problems, and providing treatment. Prompt recognition and management of adolescent depressive disorders in Morocco will reduce the risk of short- and long-term adverse outcomes and establish the basis of a healthier and egalitarian society.

### What is known about this topic

- *Adolescence is a time of increased vulnerability to depression;*
- *Sleep disturbances are closely related to psychopathology in adolescents;*
- *Adolescent depression leads to poorer educational and health outcomes*.

### What this study adds

- *In young Moroccans, close to 1/2 and 1/3 report depressive symptoms and excessive daytime sleepiness, respectively;*
- *Female gender and having divorced parents were associated with an escalation in depression symptoms;*
- *Routine screening for depression and its associated factors should be central to any preventive strategies*.
